# Cardioembolic and Small Vessel Disease Stroke Show Differences in Associations between Systemic C3 Levels and Outcome

**DOI:** 10.1371/journal.pone.0072133

**Published:** 2013-08-20

**Authors:** Anna Stokowska, Sandra Olsson, Lukas Holmegaard, Katarina Jood, Christian Blomstrand, Christina Jern, Marcela Pekna

**Affiliations:** Institute of Neuroscience and Physiology, Department of Clinical Neuroscience and Rehabilitation, The Sahlgrenska Academy at University of Gothenburg, Gothenburg, Sweden; University of Leicester, United Kingdom

## Abstract

**Background:**

Activation of the complement system has been proposed to play a role in the pathophysiology of stroke. As the specific involvement of the complement proteins may be influenced by stroke etiology, we compared plasma C3 and C3a levels in patients with cardioembolic (CE) and small vessel disease (SVD) subtypes of ischemic stroke and control subjects and evaluated their association to outcome at three months and two years.

**Methodology/Principal Findings:**

Plasma C3 and C3a levels in 79 CE and 79 SVD stroke patients, sampled within 10 days and at three months after stroke, and age- and sex-matched control subjects from The Sahlgrenska Academy Study on Ischemic Stroke were measured by ELISA. Functional outcome was assesed with modified Rankin Scale. In the CE group, plasma C3 levels were elevated only in the acute phase, whereas C3a was elevated at both time points. The follow-up phase plasma C3 levels in the upper third were associated with an increased risk of unfavorable outcome at three months (OR 7.12, CI 1.72–29.46, P = 0.007) as well as after two years (OR 8.25, CI 1.61–42.28, P = 0.011) after stroke. These associations withstand adjustment for age and sex. Conversely, three-month follow-up plasma C3a/C3 level ratios in the middle third were associated with favorable outcome after two years both in the univariate analysis (OR 0.19, CI 0.05–0.82, P = 0.026) and after adjustment for age and sex (OR 0.19, CI 0.04–0.88, P = 0.033). In the SVD group, plasma C3 and C3a levels were elevated at both time points but showed no significant associations with outcome.

**Conclusions:**

Plasma C3 and C3a levels are elevated after CE and SVD stroke but show associations with outcome only in CE stroke.

## Introduction

A growing body of evidence, largely derived from animal studies, suggests that the immune system and inflammation are involved in all stages of ischemic stroke from the intravascular response to the interruption of the blood supply through the processes in brain parenchyma leading to tissue damage to the subsequent repair and stroke-induced neural plasticity [1]. In humans, systemic levels of several inflammatory markers are altered during the first seven days after ischemic stroke and are independently related to clinical outcome scores [2].

The complement system is a major component of innate immunity that has multiple functions in the healthy as well as diseased central nervous system, ranging from elimination of synapses from maturing and axotomized neurons [3], [4], regulation of neurogenesis [5], [6] and neuroprotection [7]–[9], to tissue damage [10]–[15], and neurodegeneration [3], [16], [17].

The involvement of the complement system in the course of ischemic stroke in humans is supported by findings of local deposits of various components of complement cascade in the post-stroke brain tissue [18], [19]. Deficiency of mannose-binding lectin, an activator of the complement cascade, is neuroprotective in experimental stroke in mice and associated with favorable outcome in ischemic stroke patients [20], [21].

Systemic activation of the complement system takes place in connection with an ischemic brain insult and elevated blood levels of C3, C3a, C4d and C5-9b have been found in patients in an early post-stroke phase [22]–[24]. In addition, genetic variation in the C3 gene was found to be associated with ischemic stroke, in particular with the cryptogenic stroke subtype [25]. Although the clinical relevance and implications of these findings have received only a limited attention thus far, the extent of complement activation may play a role in determining the outcome. *E.g.* levels of soluble C5b-9 were shown to positively correlate with the clinical severity of stroke, the degree of neurological deficit and functional disability 6 days after admission [23]. Low systemic levels of mannose-binding lectin were associated with smaller infarction size and favorable outcome in ischemic stroke patients [21] and high plasma C3 levels at three months after stroke due to large vessel disease (LVD) were associated with unfavorable outcome at this time point and after two years post-stroke [26]. These findings point to the potential usefulness of systemic levels of complement proteins as predictors of outcome after ischemic stroke.

Most of the studies that examined systemic complement activation after stroke used only early time point of evaluation, involved small patients groups, and did not discriminate between etiologic stroke subtypes. As ischemic stroke is a heterogeneous disease, the significance and effects of inflammatory mediator elevation in the systemic circulation may depend on the underlying pathophysiological processes. Therefore, it is important to consider the etiology of stroke as well as analyze samples obtained both in the acute and delayed phase after ischemic stroke when studying the potential relevance of the inflammatory response in the systemic circulation for outcome. We have recently shown that the predictive value of plasma levels of C3 and C3a differ between cryptogenic and LVD stroke patients [26].

In the present study, we compared systemic levels of C3 and its activation product C3a in control subjects and in patients with CE and SVD stroke. We investigated association of plasma C3 and C3a levels to stroke, correlations to C-reactive protein (hsCRP), and associations with functional outcome at three months and two years post admission.

## Subjects and Methods

### Study Population

The study population comprises participants in the Sahlgrenska Academy Study on Ischemic Stroke (SAHLSIS) and population-based controls free from stroke and/or clinical atherothrombotic disease [26], [27]. In short, patients who presented with first-ever or recurrent acute ischemic stroke before reaching the age of 70 years were consecutively recruited (n = 600). Healthy community controls (n = 600) were randomly selected as described [26], [27], and matched to the patients based on age, sex and geographical residence, so as to achieve demographic distribution comparable to the patient group. The patients were classified into stroke subtypes according to the Trial of Org 10172 in Acute Stroke Treatment (TOAST) criteria [28], summarized in Table 1. Maximum stroke severity during the first 10 days after the stroke was scored using the Scandinavian Stroke Scale (SSS) (ranging from 0 to 58, where score 58 means no impairment). Functional outcome after three months and two years was assessed with the modified Rankin Scale (mRS).

**Table 1:** Features of TOAST classification of ischemic stroke subtypes.

Features	LVD stroke	SVD stroke	CE stroke	Other cause	Cryptogenic stroke
**Clinical**					
Cortical or cerebellar dysfunction	+	−	+	+/−	+/−
Lacnar syndrome	−	+	−	+/−	+/−
**Imaging**					
Cortical, cerebellar, brainstem, or subcortical infarct >15 mm	+	−	+	+/−	+/−
Subcortical or brainstem infarct <15 mm	−	+	−	+/−	−
**Tests**					
Stenosis of an appropriate extracranial or intracranial artery	+	−	−	−	−
Cardiac source of emboli	−	−	+	−	−
Other abnormality on tests	−	−	−	+	−

Adapted from Adams and colleagues [28].

LVD – large vessel disease; SVD – small vessel disease; CE- cardioembolic.

In the present study, we investigated ischemic stroke of CE origin and ischemic stroke due to SVD. Number of patients was intended to be similar to those used in our previous study [26], in which the number of LVD stroke patients was a limiting factor. To this end, 79 CE and 79 SVD stroke patients without missing data on three-month outcome measure were included. To minimize the effect of uneven distribution of outcome categories among the patients in each of the subgroups, and thus to improve the statistical power in the outcome regression analysis, all patients with mRS score >2 were included. The remaining ones were selected so that they represented an even distribution of the mRS scores 0, 1 and 2, *i.e.* a similar number of patients with each score was randomely selected. For each of the two groups, 40 control subjects of approximatelly the same mean age and sex distribution as the cases were selected for the analysis. These control groups were selected from controls originally matched to cases with CE stroke and SVD, and thus the present control group consisted of individuals different from those investigated in our previous study [26].

The study was approved by the Ethical Committee of the University of Gothenburg (permit number: Ö 469-99, T 553-03). All potential participants could decline to participate and all consenting patients could at any time withdraw participation. This did not in any way influence their treatment or care. All participants provided written informed consent prior to enrollment. For those participants who were unable to communicate, consent was obtained from the next-of-kin.

### Blood Sampling

In patients, blood sampling was performed within 10 days of the stroke event (median day of sampling was 4 after the stroke event, for both CE and SVD group) and at the three-month follow up. In controls, blood sampling was performed once. Venous blood was collected in ethylenediaminetetraacetic acid (EDTA-Vacuette tubes, Greiner) between 8∶30 and 10∶30 AM after an overnight fast, centrifuged and supernatants were stored at −80°C. Serum levels of high sensitivity CRP (hsCRP) were analysed as reported previously [29].

### C3 and C3a Measurements

Plasma C3 was measured by sandwich ELISA as described previously [26], with a modification of the detection antibody such that horsesradish peroxidase-conjugated polyclonal sheep anti-C3c antibody (dilution 1∶5000, Biogenesis, Poole, UK) was used. Plasma samples were analyzed for C3a by commercial ELISA kit (Quidel Corporation, San Diego, CA, USA) according to manufacturer’s recommendations but with a modified standard dilution. Inter-assay (between plates) coefficient of variation was 7.66% and 4.50%, while the mean intra-assay variation (between replicates) was 2.60% and 3.03% for the C3 and C3a assays, respectively. All quantifications were performed by an investigator blinded to any patient data. As the C3 ELISA detects the uncleaved C3 as well as the C3b and C3c fragments generated through C3 activation, C3a/C3 ratio was calculated as an additional indicator of the degree to which the plasma C3 was proteolytically activated.

### Statistical Analysis

Due to the skewness of distribution of C3 and C3a values, non-parametric tests were employed for the statistical analysis. Differences in characteristics between cases and controls were examined with the *χ* ^2^ test for proportions for categorical variables and the Mann-Whitney *U* test for continuous variables. Plasma levels of C3, C3a and hsCRP are presented as medians and inter-quartile ranges. Time-point differences of C3, C3a and hsCRP levels were compared using Wilcoxon Signed Rank test. Correlations between plasma levels of C3, C3a, serum levels of hsCRP and the SSS score were estimated by Spearman’s rank correlation coefficient, with a two-tailed significance test. For the regression analysis of the association with stroke, values for the C3 and C3a levels were standardised using mean and standard deviation of the respective control population. Furthermore, the values of complement protein levels were divided into tertiles with the cut-offs calculated for datasets of C3 or C3a measurements, obtained by pooling values for patients and controls for the respective subtype group. This procedure was performed separately for each time point and each component.

The association between complement levels and case/control status was investigated with binary logistic regression adjusted for the established risk factors: age, sex, hypertension, smoking status, diabetes mellitus and hyperlipidemia (Model 1). For studying the association of complement levels and functional outcome at three months and two years after stroke (favorable, mRS 0–2 versus unfavorable, mRS 3–6), a regression model adjusted for age and sex (Model 2) was used. ORs and 95% CIs were calculated separately for the two ischemic stroke subtypes, where patient groups were compared to their respective control group.

Statistical analysis was performed in R statistical package 2.10.1. Adjustment for multiple testing was not conducted as our study was considered to be hypothesis generating.

### Missing Values

The numbers of individuals with missing data were as follows: diabetes melitus, n = 1; hyperlipidemia, n = 6; SSS score, n = 3; hsCRP, n = 5 (acute, n = 1; follow-up, n = 4); C3, n = 6 (acute, n = 1; follow-up n = 5); C3a, n = 7 (acute, n = 1; follow-up, n = 6) and mRS, n = 2 (at two years, n = 2). Due to negligible number of missing values in the categorical variables, no adjustment for missingness was employed in the regression analysis. Missing values among continuous variables were not substituted in any of the analyses.

## Results

Demographic and clinical characteristics for the patient and control groups whose samples were used for the analysis of C3 and C3a levels are summarized in Table 2. For both groups, smoking was more common among patients than controls. Hypertension was more common in SVD patients than in the corresponding control group and hyperlipidemia occurred more often among CE patients than their controls. Both patient groups also displayed higher acute phase levels of hsCRP as compared to the controls. Additionally, acute levels of hsCRP among CE patients were markedly higher than those of SVD individuals but declined significantly at three-month follow-up (Table 2).

**Table 2:** Demographic and clinical characteristics of the study population.

	CE stroke		SVD stroke	
	Controls (n = 40)	Patients (n = 79)	Controls (n = 40)	Patients (n = 79)
Median age, years (IQ-R)	60 (55–62)	60 (55–64)	59 (57–62)	61 (57–63)
Male sex, n (%)	24 (60)	56 (71)	30 (75)	53 (67)
Hypertension, n (%)	16 (40)	42 (53)	15 (38)	57 (72)*** #
Diabetes mellitus, n (%)	3 (8)	16 (20)	3 (8)	15 (19)
Current smoking, n (%)	6 (15)	28 (35)*	5 (13)	33 (42)**
Hyperlipidemia, n (%)	22 (55)	60 (76)*	30 (75)	54 (68)
BMI, median (IQ-R)	25.84 (24.7–26.8)	25.97 (24.0–27.9)	25.57 (24.6–27.8)	27.26 (25.2–28.2)
SSS acute, median (IQ-R)		54 (47–55)		55 (52–56) #
hsCRP [mg/L],				
median (IQ-R) acute	1.94 (1.01–2.79)	6.52 (3.60–10.83)***	1.71 (1.27–2.67)	2.73 (1.59–4.34)* ###
follow-up		2.74 (1.6–4.21)&&&		2.50 (1.45–3.63)

Differences between cases and controls were examined with χ^2^ test for proportions or Mann-Whitney U or Wilcoxon signed ranked test for continuous variables.

* P<0.05;

** P<0.01;

*** P<0.001 for comparisons against controls;

# P<0.05;

### P<0.001 for comparisons against CE subtype;

&&& P<0.001 for comparisons against the acute phase. CE- cardioembolic; SVD - small vessel disease; IQ-R – inter-quartile range; BMI – body mass index; SSS – Scandinavian Stroke Scale score; hsCRP – high sensitivity C-reactive protein.

### Plasma C3 and C3a Levels after Ischemic Stroke

**Figure 1:**
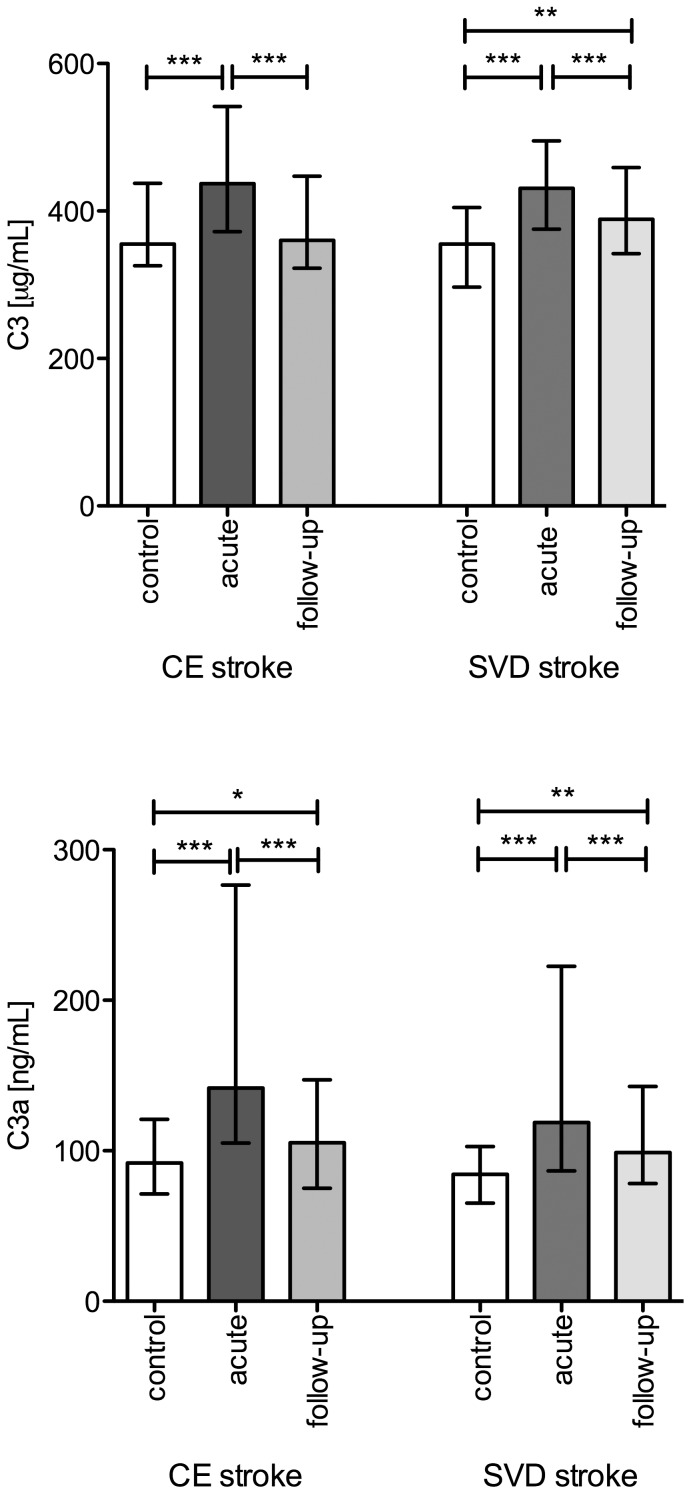
Median levels of plasma C3 (A) and C3a (B) with error bars representing inter-quartile ranges. Differences between control versus acute or follow-up measurements were examined by Mann-Whitney *U* test and acute versus follow-up by Wilcoxon signed rank test. * *P<*0.05; ** *P*<0.01; *** *P*<0.001.

In both CE and SVD stroke groups, acute phase plasma C3 levels were significantly elevated as compared to the control groups (P<0.001) as well as in comparison to the three-month follow-up C3 values (P<0.001). In addition, follow-up values of plasma C3 in SVD but not CE patients remained above the control levels (P<0.01) (Fig. 1A). Both stroke subtypes were characterized by higher plasma C3a levels compared to the respective control group at both evaluation time points (P<0.05 to P<0.001), although C3a levels in the acute phase were markedly higher than at three months after stroke (P<0.001) (Fig. 1B). There were no significant differences in C3 or C3a levels between the two patient groups or between the control groups.

Plasma C3 levels of the CE patients were at both time points positively correlated to hsCRP. In this group, plasma C3a levels in the acute phase correlated to acute phase C3 and acute hsCRP levels as well as C3a levels in the follow-up phase. In contrast, no significant correlation between the levels of complement proteins and hsCRP was observed in the SVD group. However, in this patient group we found a positive correlation between acute and follow-up levels for both C3 and C3a and the levels of C3a correlated with those of C3 at both time points (Table 3).

**Table 3:** Correlations between plasma levels of C3 and C3a, hsCRP, and SSS.

	C3afollow-up	C3 acute	hsCRP§	SSS acute
**CE stroke**				
C3 acute	–	–	0.253*	−0.313**
C3 follow-up	0.195	0.460***	0.366**	−0.297*
C3a acute	0.533***	0.248*	0.281*	−0.080
C3a follow-up	–	–	0.129	−0.153
**SVD stroke**				
C3 acute	–	–	0.232	0.214
C3 follow-up	0.240*	0.596***	0.211	0.177
C3a acute	0.438***	0.508***	−0.002	0.166
C3a follow-up	–	–	−0.121	0.137
**Controls**				
C3	–	–	0.192	–
C3a	–	0.120	−0.059	–

Values represent Spearman’s correlation coefficients (ρ) with significance levels denoted as follows:

* *P<*0.05;

** *P*<0.01;

*** *P*<0.001.

§ variable measured at the same time point as the respective indicated measurements of C3 or C3a.

–indicates non-relevant correlation.

hsCRP – high sensitivity C-reactive protein; SSS – Scandinavian Stroke Scale score; CE- cardioembolic; SVD – small vessel disease.

As expected, SVD stroke survivors presented significantly higher median SSS score than CE patients (Table 2), indicating less severe initial impairment in the SVD group. However, the difference was small, which is due to the fact that all patients with mRS >2 were selected for this study. SSS score showed a correlation with plasma C3 levels in the CE but not SVD patients (Table 3). Also only in the CE subtype, SSS scores correlated negatively with hsCRP levels measured in the acute phase as well as at 3 months after ischemic stroke (ρ = −0.510, P<0.001 and ρ = −0.306, P<0.01, respectively).

### Association of Plasma C3 and C3a Levels with Stroke

Detailed results of binary logistic regression analysis of the case/control status are presented in Table 4 and values of cut-offs for C3 and C3a tertiles are listed in Table 5. Acute phase plasma C3 concentration in the upper third was associated with stroke in the CE group both in the univariate and multivariate analysis (adjusted for age, sex, smoking status, hypertension, diabetes and hyperlipidemia) whereas C3 values in the middle third were associated with stroke only after adjustment for the risk factors. In the SVD group, acute phase plasma C3 levels in the upper and middle third were associated with stroke in both models. Follow-up plasma C3 levels in the upper third were associated with stroke in the SVD group only. The latter association remained significant also after adjustment for the traditional risk factors.

**Table 4:** Univariate and multivariate ORs with 95% CIs of association between C3 and C3a levels and stroke.

Tertiles of C3 and C3a	Controls, n	Cases, n	Univariate OR (95% CI)	Multivariate Model 1 OR (95% CI)
**CE stroke**				
C3 acute (all)	(40)	(79)		
Lower third	22	18	1.0 (reference)	1.0 (reference)
Middle third	9	26	2.21 (0.89–5.49)	3.45 (1.18–10.05)*
Upper third	9	31	6.08 (2.09–17.69)***	7.36 (2.18–24.84)***
C3 follow-up (all)	(40)	(77)		
Lower third	21	19	1.0 (reference)	1.0 (reference)
Middle third	9	28	0.80 (0.32–2.02)	0.72 (0.25–2.09)
Upper third	10	30	1.125 (0.43–2.91)	0.80 (0.28–2.37)
C3a acute (all)	(40)	(79)		
Lower third	21	19	1.0 (reference)	1.0 (reference)
Middle third	13	26	2.75 (1.09–6.92)*	3.29 (1.12–9.54)*
Upper third	6	30	6.72 (2.31–19.59)***	8.06 (2.43–26.69)***
C3a follow-up (all)	(40)	(77)		
Lower third	16	24	1.0 (reference)	1.0 (reference)
Middle third	12	26	1.24 (0.50–3.09)	1.93 (0.63–5.88)
Upper third	12	27	2.02 (0.77–5.27)	3.01 (0.99–9.13)
**SVD stroke**				
C3 acute (all)	(40)	(78)		
Lower third	19	19	1.0 (reference)	1.0 (reference)
Middle third	14	23	3.11 (1.22–7.93)*	3.24 (1.10–9.54)*
Upper third	6	29	5.76 (2.06–16.08)***	6.39 (1.94–21.02)**
C3 follow-up (all)	(40)	(65)		
Lower third	19	19	1.0 (reference)	1.0 (reference)
Middle third	10	19	1.83 (0.73–4.58)	1.88 (0.66–5.42)
Upper third	10	27	3.68 (1.36–10.00)*	3.47 (1.11–10.90)*
C3a acute (all)	(40)	(77)		
Lower third	23	15	1.0 (reference)	1.0 (reference)
Middle third	12	25	2.16 (0.88–5.32)	1.94 (0.65–5.78)
Upper third	4	31	25.71 (5.43–121.6)***	41.51 (6.81–252.8)***
C3a follow-up (all)	(40)	(65)		
Lower third	19	19	1.0 (reference)	1.0 (reference)
Middle third	12	22	0.95 (0.39–2.35)	0.65 (0.22–1.88)
Upper third	8	23	4.12 (1.40–12.10)**	3.51 (1.02–12.04)*

OR – odds ratio; CI – confidence intervals; SVD – small vessel disease, CE – cardioembolic.

Model 1: adjusted for age, sex, hypertension, smoking, diabetes and hyperlipidemia;

* *P<*0.05;

** *P*<0.01;

*** *P*<0.001.

**Table 5:** Cut-offs for tertiles of plasma C3 and C3a levels.

	CE stroke		SVD stroke	
	acute	follow-up	acute	follow-up
C3 (mg/L)				
Lower third	<370.55	<337.84	<366.97	<346.49
Middle third	370.55–460.42	337.84–409.14	366.97–436.59	346.49–411.50
Upper third	>460.42	>409.14	>436.59	>411.5
C3a (µg/L)				
Lower third	<95.01	<82.86	<85.50	<81.03
Middle third	95.01–162.31	82.86–120.81	85.50–123.82	81.03–107.96
Upper third	>162.31	>120.81	>123.82	>107.96

CE – cardioembolic; SVD – small vessel disease.

Higher acute C3a levels (upper third for SVD and upper and middle third for CE) were also associated with stroke under the unadjusted as well as the multiple-adjusted model. At three months after stroke, elevated C3a plasma concentration was associated with case/control status only in the SVD group. This association was independent of traditional risk factors.

### Association of Plasma C3 and C3a Levels with Functional Outcome

Among the CE stroke patients, 22 (28%) and 20 (26%) had an unfavorable outcome (mRS 3–6) at three months and at two years after the stroke event, respectively. In the SVD stroke group, 9 (11%) and 8 patients (12%) had an unfavorable outcome at three-month and two-year follow-up, respectively. The follow-up phase plasma C3 levels in the upper third were found to be associated with an increased risk of unfavorable outcome at three months as well as two years after stroke in the CE group. These associations withstand adjustment for age and sex. Interestingly, in the same group, three-month follow-up plasma C3a/C3 level ratios in the middle third were associated with favorable outcome after two years both in the univariate analysis and after adjustment for age and sex (Table 6) As expected, low SSS score was a highly predictive of poor outcome in all the analyses in both patient groups (P<0.01). The associations between the follow-up plasma C3 levels in the upper third and unfavorable outcome at three months and two years remained significant even after the inclusion of SSS score in the multivariate analysis (OR 10.28, CI 1.19–89.20, P = 0.035 and OR 9.36, CI 1.01–86.70, P = 0.048, respectively). Similarly, the associations between the C3a/C3 level ratio in the middle third at three-month follow-up and outcome after two years withstood the adjustment for SSS score (OR 0.45, CI 0.003–0.680, P = 0.026).

**Table 6:** Selected§ univariate and multivariate ORs with 95% CIs of association between C3 and C3a levels and functional outcome.

Tertiles of C3 and C3a	Favorableoutcome, n	Unfavorableoutcome, n	UnivariateOR (95% CI)	Multivariate Model 2OR (95% CI)
*Outcome at three months*				
**CE stroke**				
C3 follow-up (all)	(55)	(22)		
Lower third	23	3	1.0 (reference)	1.0 (reference)
Middle third	18	6	2.56 (0.56–11.65)	2.13 (0.45–10.03)
Upper third	14	13	7.12 (1.72–29.46)**	8.72 (1.99–38.24)**
C3a/C3 follow-up (all)	(55)	(22)		
Lower third	15	9	1.0 (reference)	1.0 (reference)
Middle third	21	4	3.14 (0.30–32.65)	3.38 (0.31–36.31)
Upper third	19	9	4.78 (0.52–44.25)	4.52 (0.47–43.05)
*Outcome at two years*				
**CE stroke**				
C3 follow-up (all)	(57)	(20)		
Lower third	24	2	1.0 (reference)	1.0 (reference)
Middle third	17	7	4.94 (0.91–26.77)	4.49 (0.79–25.49)
Upper third	16	11	8.25(1.61–42.28)*	9.11 (1.70–48.82)**
C3a/C3 follow-up (all)	(57)	(20)		
Lower third	14	10	1.0 (reference)	1.0 (reference)
Middle third	22	3	0.19 (0.05–0.82)*	0.19 (0.04–0.88)*
Upper third	21	7	0.47 (0.14–1.25)	0.44 (0.13–1.51)

§ - remaining non-significant results are not shown;

* *P<*0.05,

** *P*<0.01;

OR - odds ratio; CI - confidence interval; CE- cardioembolic.

Model 2: adjusted for age and sex.

In a univariate analysis, the hsCRP levels also showed an association with outcome in the CE group such that the acute hsCRP levels were associated with unfavorable outcome at three months and two years (OR 1.03, CI 1.01–1.06, P = 0.012 and OR 1.02, CI 1.001–1.040, P = 0.039, respectively). Also, the follow-up hsCRP levels were associated with unfavorable outcome at two years (OR 1.11, CI 1.02–1.21, P = 0.017). For that reason, we assessed the effect of hsCRP levels on the associations between plasma C3 levels and C3a/C3 level ratio and outcome in this patient group. The associations between the follow-up plasma C3 levels in the upper third and unfavorable outcome at three months and two years remained significant after the inclusion of acute hsCRP in the multivariate analysis (OR 12.12, CI 2.34–62.75, P = 0.003 and OR 10.90, CI 1.87–63.59, P = 0.008, respectively). The associations between the follow-up plasma C3 levels in the upper third and unfavorable outcome at three months and two years remained significant even after the inclusion of follow-up hsCRP levels in the multivariate analysis (OR 7.64, CI 1.70–34.32, P = 0.008 and OR 7.84, CI 1.39 to 44.16, P = 0.002, respectively). Similarly, the association between the C3a/C3 level ratio in the middle third at three-month follow-up and outcome after two years withstands the adjustment for acute as well as follow-up hsCRP levels (OR 0.20, CI 0.04 to 0.89, P = 0.035 and OR 0.19, CI 0.04 to 0.95, P = 0.043, respectively). Interestingly, in the multivariate analysis using age, sex, acute hsCRP and follow-up C3 or C3a/C3 ratio as covariates, hsCRP did no longer show an association with outcome at two years. Further, in the multivariate analysis using age, sex and follow-up hsCRP and C3 as covariates, the association between hsCRP and outcome at two years was lost.

In the SVD group, no associations between outcome and any of the complement-related measurements or hsCRP were found.

## Discussion

Despite the growing evidence for the importance of the complement system in the normal as well as diseased brain, there are only a few studies investigating the changes in complement proteins and their activation product levels in the systemic circulation of ischemic stroke patients [20]–[23], [26], [30] and in particular the relevance of these alterations for short and long term outcome [20], [21], [23], [26]. As it is conceivable that subtype-specific pathophysiologies could result in a uniqe profile of systemic levels of markers and because stroke subtypes differ in severity of the resulting impairment and risk for secondary complications, studying an unstratified population of stroke patients may preclude identification of potential clinically usefull associations, unless a very large sample size is available. Therefore, the heterogeneity of ischemic stroke etiologies needs to be taken into consideration when assessing the clinical significance of circulating biomarkers and their prognostic value.

Here, we have found that both in CE and SVD stroke, plasma levels of both C3 and C3a were elevated in the acute phase and were associated with ischemic stroke also after adjustment for the influence of traditional risk factors. These results confirm our previous findings from the LVD and cryptogenic ischemic stroke subtypes [26] and further support the notion that complement activation in the early post-stroke phase contributes to the systemic inflammatory response triggered by brain ischemia. In the SVD but not CE subtype, C3 levels remained elevated also three months after stroke and high plasma C3 levels at this time point showed an association with patient status independently of traditional risk factors. We interpret the high plasma C3 levels in the delayed phase after SVD stroke as an indicator of C3 levels being elevated already prior to the insult. These results corroborate our previous findings from the LVD subtype [26] and point to similarities between SVD and LVD, the chronic nature of the underlying pathology and the potential role of complement in this process. Indeed, atherosclerosis is often associated also with small vessel disease stroke [31] and animal [32]–[37] as well as clinical studies [38]–[44] demonstrate that several stages of atherogenesis are regulated by the complement system and in particular C3. Although the follow-up C3a levels were elevated in both patient groups, C3a levels were associated with ischemic stroke only in the SVD group at this time point. This discrepancy and the possible differences in the mechanisms leading to continuous C3a generation in the two patient groups merit further investigation.

The CE and SVD stroke subtypes showed clear differences in the correlation of systemic C3 and C3a levels with the severity of impairment and association with outcome. In the CE group, high C3 levels at follow-up were associated with increased risk of unfavorable outcome at both three months and two years after stroke. There was a positive correlation between acute and follow-up C3 levels in this group and positive correlation between plasma C3 levels in the acute phase and SSS score. As direct infarct volume evaluation was not performed in our study, the SSS score, which shows a strong correlation with both short and long-term outcome, was used as an indirect measure of infarct volume. Further, as shown here as well as in other studies [30], [45], systemic CRP levels within the first week after stroke are associated with long–term outcome and correlate with brain infarct volume. Thus, the association between systemic C3 response and outcome after CE stroke could simply be explained by the extent of damage and impairment. However, the observed association between C3 levels and outcome within CE subtype remained also after adjustment of the regression analysis for SSS score or hsCRP levels and the results of multivariate analysis show that follow-up plasma C3 levels may be a better predictor of long-term outcome than hsCRP. Thus, at least in the delayed phase after CE stroke, the systemic levels of C3 could be a useful parameter, in addition to or instead of hsCRP, in predicting the long-term outcome.

It is noteworthy that additional factors, such as *e.g.* infection or subsequent more subtle thromboembolic events that can contribute to high systemic C3 levels and negative outcome in the CE patients, could have also affected the results. As the SAHLSIS project was not initially designed to study inflammatory responses, a detailed record of recent infections or chronic inflammatory events is missing. The lack of such information is a clear limitation of our study. Indeed, plasma C3a levels, which correspond directly to the extent of complement activation, did not show any correlation with SSS score in any of the ischemic stroke subtypes examined [26]. Thus, in addition to the extent of tissue damage, systemic activation of the complement system appears to be influenced by other factors such as the underlying pathophysiological processes and infections. Further investigation in larger studies that would control for the effect of infections could help clarifying this matter.

Unexpectedly, in the CE subtype plasma C3a/C3 level ratio in the middle third (indicating some degree of systemic activation of the complement cascade) at three-month follow-up was associated with favorable outcome after two years. Although interesting, this observation might have simply occurred due to low numbers of individuals with unfavorable outcome that were classified into this C3a/C3 level range (middle third). Of note is, however, that this association remained significant even after adjusting for SSS score or hsCRP levels. This intriquing finding requires further investigation in a larger cohort as well as warrants mechanistic studies on the benefial effects of complement activation, in particular the atheroprotective effects of the C3a derivative C3a_desArg_ [37], [46]. There was no association between C3 or C3a levels with outcome in the SVD group. Given the per definition small infarct volume that yields a narrow distribution of SSS and mRS values in this ischemic stroke subtype, this finding is not surprising. Indeed, the two patient groups studied showed a small but significant difference in the initial impairment. Thus, we cannot fully rule out the possibility that the differences in outcome and other parameters used for the comparisson are not, at least partly, due to differences in the severity of injury and impairment between the CE and SVD stroke subtypes.

The two patient groups were also different with regard to the correlation of plasma C3 and C3a levels with hsCRP. In the CE group, hsCRP showed a positive correlation with both C3 and C3a in the acute phase and the positive correlation between hsCRP and C3 in this group persisted at the three-month follow-up. No such correlation was seen in the SVD group. Although both C3 and CRP are acute phase proteins, the liver synthesis of which is dramatically increased in response to injury and both are increased after stroke, their systemic levels are regulated by diverse mechanisms that might be differently affected by the distinct ischemic stroke etiologies [26]. With regard to the correlation between systemic hsCRP and C3 levels, CE appears to resemble the cryptogenic stroke subtype [26]. In contrast to SVD, we observed a positive correlation between C3a and hsCRP levels in patients of the LVD group [26]. Thus with respect to the regulation of systemic C3, C3a and CRP, the SVD and LVD appear to be two distinct entities and CRP, C3 and C3a levels provide complementary information about the patient’s inflammatory status.

In conclusion, our data show that plasma C3 and C3a are elevated in CE and SVD stroke but the dynamics of this increase as well as the potential prognostic value of these markers is influenced by ischemic stroke etiology. Together with our previous report on cryptogenic and LVD stroke [26], these findings point to the need for future studies aiming at decifering the immune response to stroke to stratify the ischemic stroke patients not only according to stroke severity but also the importance of controling for stroke etiology [47]. As complement activation in the peripheral circulation is determined by the response to stroke as well as the underlying pathology, more studies on the role of the complement system in pathogenesis and outcome of ischemic stroke based on larger patient populations are warranted.
